# Mechanisms of metabolic stress induced cell death of human oligodendrocytes: relevance for progressive multiple sclerosis

**DOI:** 10.1186/s40478-023-01601-1

**Published:** 2023-07-05

**Authors:** Milton Guilherme Forestieri Fernandes, Abdulshakour Mohammadnia, Florian Pernin, Laura Eleonora Schmitz-Gielsdorf, Caroline Hodgins, Qiao-Ling Cui, Moein Yaqubi, Manon Blain, Jeffery Hall, Roy Dudley, Myriam Srour, Stephanie E. J. Zandee, Wendy Klement, Alexandre Prat, Jo Anne Stratton, Moses Rodriguez, Tanja Kuhlmann, Wayne Moore, Timothy E. Kennedy, Jack P. Antel

**Affiliations:** 1 Neuroimmunology Unit, Montreal Neurological Institute, Department of Neurology and Neurosurgery, McGill University, 3801 Rue University, Montreal, QC H3A 2B4 Canada; 2 Institute of Neuropathology, University Hospital Münster, Albert-Schweitzer-Campus 1, 48149 Münster, Germany; 3 Department of Neurosurgery, Department of Neurology and Neurosurgery, McGill University Health Centre, 3801 Rue University, Montreal, QC H3A 2B4 Canada; 4 Department of Pediatric Neurosurgery, Montreal Children’s Hospital, 1001 Decarie Blvd, Montreal, QC H4A 3J1 Canada; 5 Division of Pediatric Neurology, Montreal Children’s Hospital, 1001 Decarie Blvd, Montreal, QC H4A 3J1 Canada; 6 Department of Neuroscience, Faculty of Medicine, Université de Montréal, Pavillon Roger- Gaudry, 2900 Edouard Montpetit Blvd, Montreal, QC H3T 1J4 Canada; 7 Department of Neurology and Neurosurgery, McGill University, 3801 Rue University, Montreal, QC H3A 2B4 Canada; 8 Department of Neurology, Mayo Clinic Foundation, 1216 2nd St SW, Rochester, MN 55902 USA

**Keywords:** Neurodegeneration, Demyelination, Myelin, Neurodegenerative disease, Cell survival

## Abstract

Oligodendrocyte (OL) injury and loss are central features of evolving lesions in multiple sclerosis. Potential causative mechanisms of OL loss include metabolic stress within the lesion microenvironment. Here we use the injury response of primary human OLs (hOLs) to metabolic stress (reduced glucose/nutrients) in vitro to help define the basis for the in situ features of OLs in cases of MS. Under metabolic stress in vitro, we detected reduction in ATP levels per cell that precede changes in survival. Autophagy was initially activated, although ATP levels were not altered by inhibitors (chloroquine) or activators (Torin-1). Prolonged stress resulted in autophagy failure, documented by non-fusion of autophagosomes and lysosomes. Consistent with our in vitro results, we detected higher expression of LC3, a marker of autophagosomes in OLs, in MS lesions compared to controls. Both in vitro* and *in situ, we observe a reduction in nuclear size of remaining OLs. Prolonged stress resulted in increased ROS and cleavage of spectrin, a target of Ca^2+^-dependent proteases. Cell death was however not prevented by inhibitors of ferroptosis or MPT-driven necrosis, the regulated cell death (RCD) pathways most likely to be activated by metabolic stress. hOLs have decreased expression of VDAC1, VDAC2, and of genes regulating iron accumulation and cyclophilin. RNA sequencing analyses did not identify activation of these RCD pathways in vitro or in MS cases. We conclude that this distinct response of hOLs, including resistance to RCD, reflects the combined impact of autophagy failure, increased ROS, and calcium influx, resulting in metabolic collapse and degeneration of cellular structural integrity. Defining the basis of OL injury and death provides guidance for development of neuro-protective strategies.

**Supplementary Information:**

The online version contains supplementary material available at 10.1186/s40478-023-01601-1.

## Introduction

Demyelination within the CNS is the pathologic hallmark of multiple sclerosis (MS). Histologic analyses indicate that while the number of oligodendrocytes (OLs), the myelin producing cells, are relatively preserved in initial demyelinating white matter lesions (“relapsing phase”), there is detectible cell loss in active post-demyelinating lesions, with increasing loss in mixed active / inactive lesions (also named chronic active or smoldering lesions) [20, 25]. Actively demyelinating lesions are mostly found in patients with early relapsing remitting disease course, whereas the proportion of mixed lesions significantly increases in patients with progressive disease [3, 26, 30]. Prineas et al. reported that surviving OLs at the lesion border in chronic active white matter lesions only rarely showed apoptotic nuclei and were not TUNEL positive [38]. Bonetti and Raine also concluded that the OLs associated with MS lesions in cases of chronic progressive MS do not undergo apoptosis [4].

Mechanisms of cell death have been considered under the broad categories of either accidental or regulated cell death (RCD) [12]. Accidental cell death is the end result of an instantaneous and catastrophic process that results in a state that is incompatible with cell survival; this is typically attributed to acute exposure of cells to severe external physical insults that result in rupture of the plasma membrane and release of cytoplasm into the extracellular space [8]. In contrast, RCD implies a dedicated molecular machinery that can be modulated [12]. Multiple RCD pathways are now recognized that can also result from perturbations of the intra- or extracellular environments when adaptive responses cannot restore homeostasis [12].

Conditions implicated in OL cell loss in progressive MS include infection/inflammation and metabolic stress [12]. In a previous study we showed that primary adult human OLs, a cell type that is heavily dependent on glycolysis as an energy source [42], when challenged with metabolic stress conditions, undergo delayed cell death without activation of apoptotic pathways when compared to young pediatric brain derived OLs and especially fetal brain derived progenitor cells [7, 9]. Exposure of adult human OLs to pro-inflammatory cytokines induced only sub-lethal injury (dying back of cell processes) [6, 37]. Examining human MS lesions we detected increased expression of the integrated stress response (ISR) constituent phosphorylated EIF2α in situ, consistent with local metabolic stress. Further, RNA sequencing studies have revealed the upregulation of an array of metabolic stress related genes in OLs in MS lesions [22].

The central aim of the current study was to define the mechanistic basis of cell death of primary human OLs (hOLs) in vitro in response to metabolic stress (reduced glucose/nutrients) and relate this to the in situ features of OLs that evolve during the course of MS. Our in vitro studies using this model of metabolic stress demonstrate a significant reduction in ATP per hOL that precedes any change in cell survival. Both in the in vitro stress model and in situ MS lesions, we detect an increase in LC3 in OLs, a marker of autophagosomes, indicating that the initially activated autophagy pathway has stalled. We detected nuclear condensation and volume reduction (pyknosis) [5] in hOLs in vitro under sustained stress conditions and in remaining OLs in “active/post-demyelinating” lesions. We show that prolonged stress in vitro results in increased ROS and cleavage of spectrin, a target of Ca^2+^-dependent proteases in hOLs; however both in vitro and in situ, the hOLs resist triggering either ferroptosis or mitochondrial permeability transition-driven necrosis (MPTN), RCD pathways commonly linked to metabolic stress and operative in MS related models including EAE and cuprizone toxicity [12, 21, 23]. We consider that the distinct cell death response of hOLs, a cell type resistant to activating RCD pathways, reflects the combined impact of autophagy failure, increased ROS and calcium influx, resulting in the collapse of cellular structural integrity. The prolonged time course of hOL cell death may provide an opportunity for therapeutic targeting.

## Materials and methods

### In situ immunohistochemical studies - MS and control tissue samples

For LC-3 immuno-fluorescence-based histochemistry, human rapid post-mortem brain tissue samples were obtained from the Neuroimmunology Research Laboratory, Centre de Recherche du Centre Hospitalier de l’Université de Montréal (CRCHUM) under ethical approval number BH07.001.31. Sections with areas of chronic active demyelination were selected based on Luxol Fast Blue-Hematoxylin and Eosin (LFB/H&E) staining and presence of macrophages, some of which contained LFB positive material. The immunohistochemistry procedure and confocal imaging were performed as previously described [37]. Sudan black was added to suppress autofluorescence. Primary antibodies used were LC3 (1:500, NB100-2220 Novus Biologicals) and Nogo-A (1:5000, University of Zurich). Secondary antibodies used were goat anti-rabbit Alexa Fluor 488 (1:500) and goat anti-mouse Alexa Fluor 555 (1:500). LC3 expression was quantified by pixel intensity in individual Nogo-A + cells. Data were derived by blinded observers measuring 10–15 cells per region of interest. Results are expressed as mean pixel intensity of the cells counted in each region of interest.

For the tissue sections selected to assess OL nuclei in MS tissue sections, the immunohistochemical labeling procedures, and means of quantitating cell numbers are as detailed previously [20].

### In vitro studies - human surgical samples

Anonymized surgically resected brain tissue samples were obtained from the Department of Neuropathology at the Montreal Neurological Institute and Hospital (MNI) and from the Montreal Children’s Hospital. All had non-tumor related focal epilepsy. Data on individual samples are provided in the Supplementary table. Studies were approved by the MNI Neurosciences Research Ethics Board (Protocol ANTJ 1988/3) and the Montreal Children’s Hospital Research Ethics Board.

### Cell isolation

Normal appearing tissue was derived from “surgical corridors” resected to access sites of pathology. As previously described [9], tissue derived from CUSA bags was subjected to trypsin digestion followed by Percoll gradient centrifugation to obtain a myelin-depleted whole-cell fraction comprised mainly of OLs and microglia with few if any astrocytes or neurons. An enriched OL population was obtained by plating the total cell population overnight in culture flasks; the floating cell fraction was recovered, leaving behind adherent microglia. The final culture contains an average of ~ 90% O4 positive cells (OLs), < 5% micorglia, and only rarely astrocytes.

### Cell culture

After selection, primary human cells were plated in 96-well or 24-well plates coated with poly-lysine and extra-cellular matrix at a density of 3 × 10^4^ cells (96-wells plate) or 1 × 10^6^ cells (24-wells plate) per well. Cells were cultured in DMEM-F12 media supplemented with N1 (Sigma, Oakville, ON, Canada). For metabolic deprivation experiments, cells were cultured in DMEM containing 0.25 g/l of glucose (LG) or with no glucose added (NG). HeLa cells were cultured and treated in DMEM + 10% fetal calf serum.

### Immunocytochemistry

Cells were live stained with propidium iodide (PI; Invitrogen) (1:200) for cell viability measurements and with O4 monoclonal antibody (R&D Systems, Minneapolis, MN) (1:200) for 15 min at 37 °C and then fixed with 4% paraformaldehyde for 10 min at rt. Goat anti-mouse IgM Cy3 (1:500) was used as secondary antibody, 30 min at rt. Staining of autophagosomes and lysosomes was done as previously described [44]. Cells were washed and permabilized in 100% cold methanol for 10 min at -20 °C. Cells were incubated in blocking buffer for 30 min at rt. Labeling using primary antibodies against LAMP-1 (MA1-184, Invitrogen, mouse IgG1), LC3 (2775 S, Cell Signaling, rabbit) and alpha-II spectrin (PA5-35383, ThermoFisher, IgG rabbit) at 1:200 dilution was performed overnight at 4 °C. Cells were incubated in secondary antibodies coupled to Alexa 488 or Alexa 647 at 1:500 dilution for 2 h at rt. Cell nuclei were stained with Hoechst 33258 (1:1000) for 1 h at rt. Coverslips were mounted with Prolong gold (Invitrogen) [44]. Reagents used were: chloroquine (Sigma, Oakville, ON, Canada; 10 µM), erastin (Sigma-Aldridge, Saint Louis, MO, USA), H_2_O_2_ (Sigma, Oakville, ON, Canada), cyclosporine A (Sigma, Oakville, ON, Canada), ferrostatin-1 (Sigma-Aldridge, Saint Louis, MO, USA) and torin-1 (Selleckchem, Houston, TX, USA).

### Confocal microscopy

Images of intracellular components were obtained using a Leica TCS SP8 with a 63x/1.4 n.a. oil immersion objective at rt. LAS X was used as acquisition software, ImageJ was used for quantification and R for data and statistical analysis.

### Western blot analyses

Cellular homogenates, 5–20 µg of total protein in each sample, were resolved using 7.5% (high MW targets) or 15% (low MW targets) SDS-PAGE. Proteins were electroblotted to a nitrocellulose membrane. Membranes were blocked with 5% milk and probed with 1:5000 alpha-II spectrin polyclonal antibody (PA5-35383, ThermoFisher, IgG rabbit) and anti-Caspase 3 (31A1067 Novus Biological, IgG1 mouse). Anti-rabbit horseradish peroxidase- conjugated secondary antibody was applied and bands visualized using an ECL Western blot detection kit (Cell Signaling, Danvers, MA).

### ATP and H_2_O_2_ assays

Levels of ATP and H_2_O_2_ were measured using Cell Titer-Glo 2.0 (Promega, G9242) and ROS-Glo H_2_O_2_ Assay (Promega G8820). Normalization to cell number was calculated using the ratio between the measures of ATP and H_2_O_2_ obtained in the assay and the number of cells counted after Hoechst 33258 staining (1:1000, 1 h at rt) using ImageJ.

### Molecular studies

RNA was extracted from selected hOLs as previously described [19]. Quality control of the bulk RNA samples, as well as the library preparation, RNA-sequencing, and alignment were performed as describe in Luo et al. [31]. Raw read counts were normalized, variance-stabilized transformed and differential gene expression analysis were done using DESeq2 package in R [29]. Adjusted p-value < 0.05 and log2 fold change > 1 were used to identify DEGs. Single sample gene set enrichment analysis (ssGSEA) implanted in GenePattern [41] was used to run pathway level enrichment analysis on bulk RNA-seq data. To define reference for ssGSEA, we generated signatures for different cell death pathways derived from XDeathDB database [10]. We used Li et al., and Bauer et al., publications to define signatures of ferroptosis and MPT derived necrosis [2, 27]. In addition to our local datasets, we used five datasets for HeLa cell lines with gene accession numbers GSE188567, GSE186370, GSE155493, GSE157717, and GSE174116 obtained from gene expression omnibus (GEO) database [1, 10, 17]. Normalized read counts were used for hierarchical clustering and results were visualized in heatmaps format using GenePattern. [41]. Single nuclear RNA-seq dataset from Jakel et al. [22] was downloaded and analyzed as previously described [47].

### Statistics and reproducibility

#### In vitro studies

All statistics are presented as the mean and standard error of the mean. The statistical test used and level of significance are indicated in the figure legends.

#### Transcriptome studies

Data were analyzed using GraphPad Prism version 8.3.0. Throughout the manuscript, *p-values* are indicated in the graphs and non-significant values are shown using “ns”.

## Results

### Metabolic stress rapidly reduces ATP in hOLs followed by autophagy failure

**Fig. 1:**
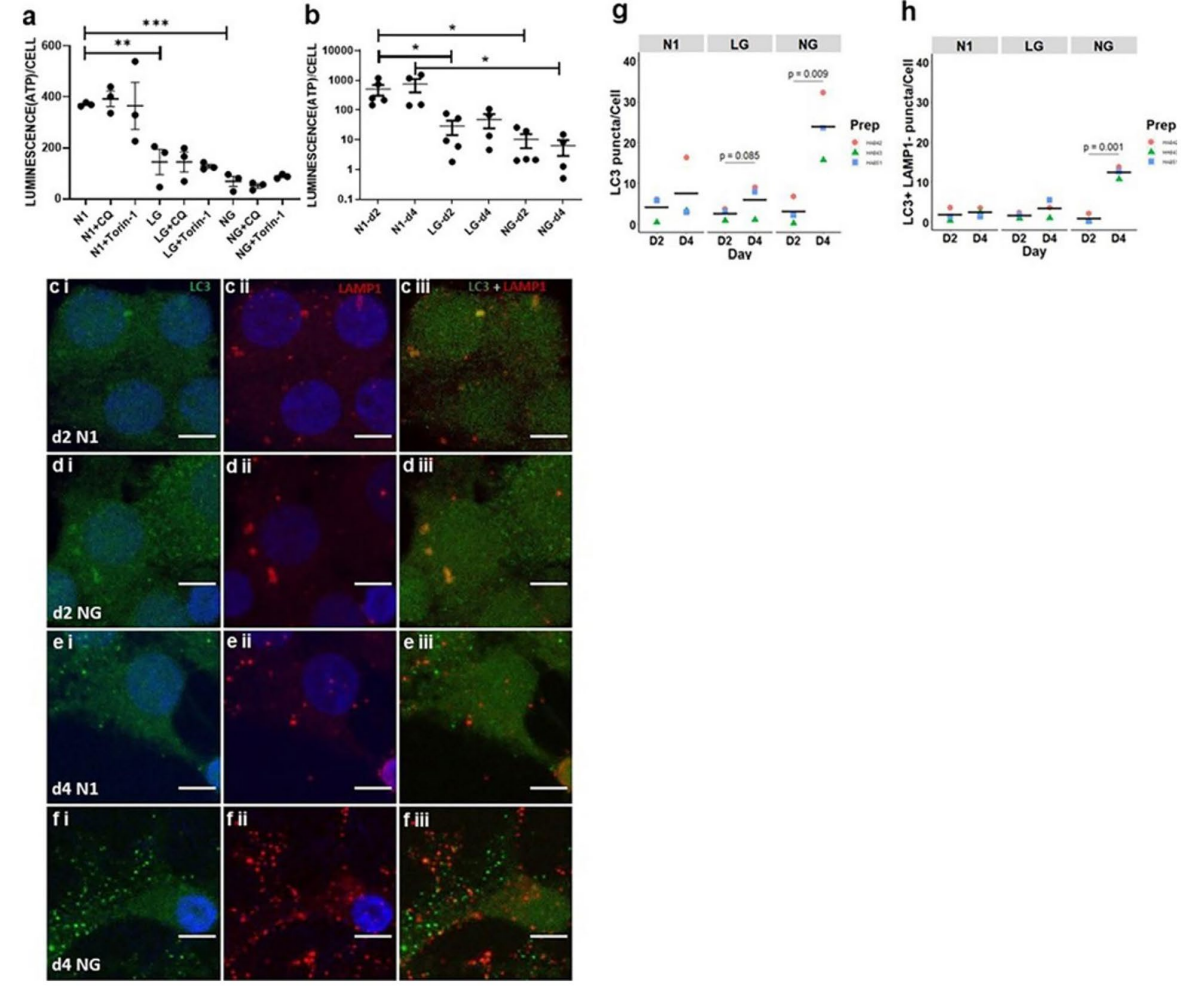
Metabolic stress reduces ATP resulting in failure of autophagic flux in hOLs. **a** ATP levels in hOLs cultured in optimal (N1), low glucose (LG), and no glucose (NG) conditions combined with chloroquine (CQ) or Torin-1. 6 h of treatment resulted in a significant decline in ATP under LG and NG conditions without an additive effect of CQ or Torin-1. Statistical significance was verified by ANOVA/Dunnett’s test: **(< 0.01), ***(< 0.001) **b** ATP levels in hOL cultured in N1, LG, and NG conditions. After 2 and 4 days of treatment, ATP levels continue to decline in LG and NG conditions. Mean ± SEM for each condition shown in the figure. Statistical significance was verified by ANOVA/Dunnett’s test: *(< 0.05). **c-f** Confocal images of autophagosomes (LC3 - green) and lysosomes (LAMP1 – red) in hOLs under control (N1) or no glucose (NG) conditions. After 2 days of treatment under N1 **c** and NG **d** conditions, few autophagosomes (LC3 puncta) were detected (**c i**, **d i)**. The majority of these autophagosomes were fused with lysosomes (LAMP1 puncta) **(c iii**, **d iii)**. **e** After 4 days of treatment under N1 conditions, few autophagosomes (LC3 puncta) were detected (**e i**). The majority of these autophagosomes were fused with lysosomes (LAMP1 puncta) (**e iii**). **f** After 4 days of treatment under NG conditions, many autophagosomes (LC3 puncta) were detected (**f i**); most were not fused with lysosomes (LAMP1 puncta) (**f iii**). **g** Quantification of autophagosomes (LC3 puncta) per cell in N1, LG and LG conditions after 2 and 4 days of treatment. After 2 days, in all conditions, the number of autophagosomes per cell was low. After 4 days, this number was slightly increased in LG conditions and strongly increased in NG conditions. Statistical significance was verified by Student’s t-test. **h** Quantification of autophagosomes not fused with lysosomes (LC3 + ve LAMP1 -ve puncta) per cell in N1, LG and NG conditions after 2 and 4 days of treatment. After 2 days, in all conditions, the number of autophagosomes not fused with lysosomes per cell was low. After 4 days, this number increased in NG conditions. Each dot-color corresponds to an independent biological sample. Bar indicates the mean. hOLs were marked with DAPI (blue), indicating the cell nucleus. Statistical significance was verified by Student’s t-test.

*Reduction of ATP levels under metabolic stress *in vitro* -* In a previous study, we showed that autophagic flux is increased in metabolically stressed hOLs and that inhibiting autophagic flux with chloroquine in metabolically stressed hOLs causes an accumulation of autophagosomes and increases cell death after 2 days [9]; while cell death was not observed under stress conditions alone. We further verify activation of AMPK, a key regulator of autophagy, in hOL under NG conditions (Supplementary Fig. 1); To determine the impact of metabolic stress on cytoplasmic ATP, we assessed ATP levels per cell under such conditions. As shown in Fig. 1a, we detected a significant decrease in the amount of ATP in hOLs within 6 h in cell culture medium containing low glucose (LG) and no glucose (NG) compared to cells in optimal culture media (N1) (Fig. 1a). The decline in ATP levels continued during the following 2 and 4 days (Fig. 1b).

*Autophagy modulation does not impact ATP levels* - To assess the contribution of autophagy to the energy status of the cells under these stress conditions, we evaluated ATP levels in the presence of chloroquine, an autophagy inhibitor, and Torin-1, an inhibitor of mTOR, thus an activator of autophagy (Fig. 1a). Neither chloroquine nor Torin-1 had a measurable impact on ATP levels under basal or stress conditions.

*Autophagy failure under metabolic stress***–** Autophagy provides cellular functions beyond energy production, including misfolded protein clearance and material recycling in the cell [45]. Autophagy itself requires ATP for the transport and fusion of autophagosomes and lysosomes [28]. Therefore, the ATP depletion detected during metabolic stress could be the underlying cause of autophagy failure. To address the status of autophagy in hOLs when challenged with metabolic stress, we used confocal microscopy with LC3 as a marker of autophagosomes and LAMP1 as a lysosomal marker [44]. After 2 days in culture, few LC3-positive vesicles (autophagosomes) were observed in N1 and NG conditions (Fig. 1c i-d i); the few LC3-positive vesicles detected were colocalized with LAMP1-positive vesicles (Fig. 1c iii-d iii) indicating the formation of autolysosomes. After 4 days, a limited number of LC3-positive vesicles were detected in the N1 condition (Fig. 1e i) and most were colocalized with LAMP1-positive vesicles (Fig. 1e iii), indicating successful fusion with lysosomes. In NG conditions, we detected a considerable increase in the number of LC3-positive vesicles (Fig. 1f i), and most were not colocalized with LAMP1-positive vesicles (Fig. 1f iii), indicating autophagy failure. Quantification revealed a significant increase in the number of LC3-positive vesicles per cell induced by metabolic stress and in the number of LC3-positive vesicles not colocalized with LAMP1 in hOLs (Fig. 1g, h).

**Fig. 2:**
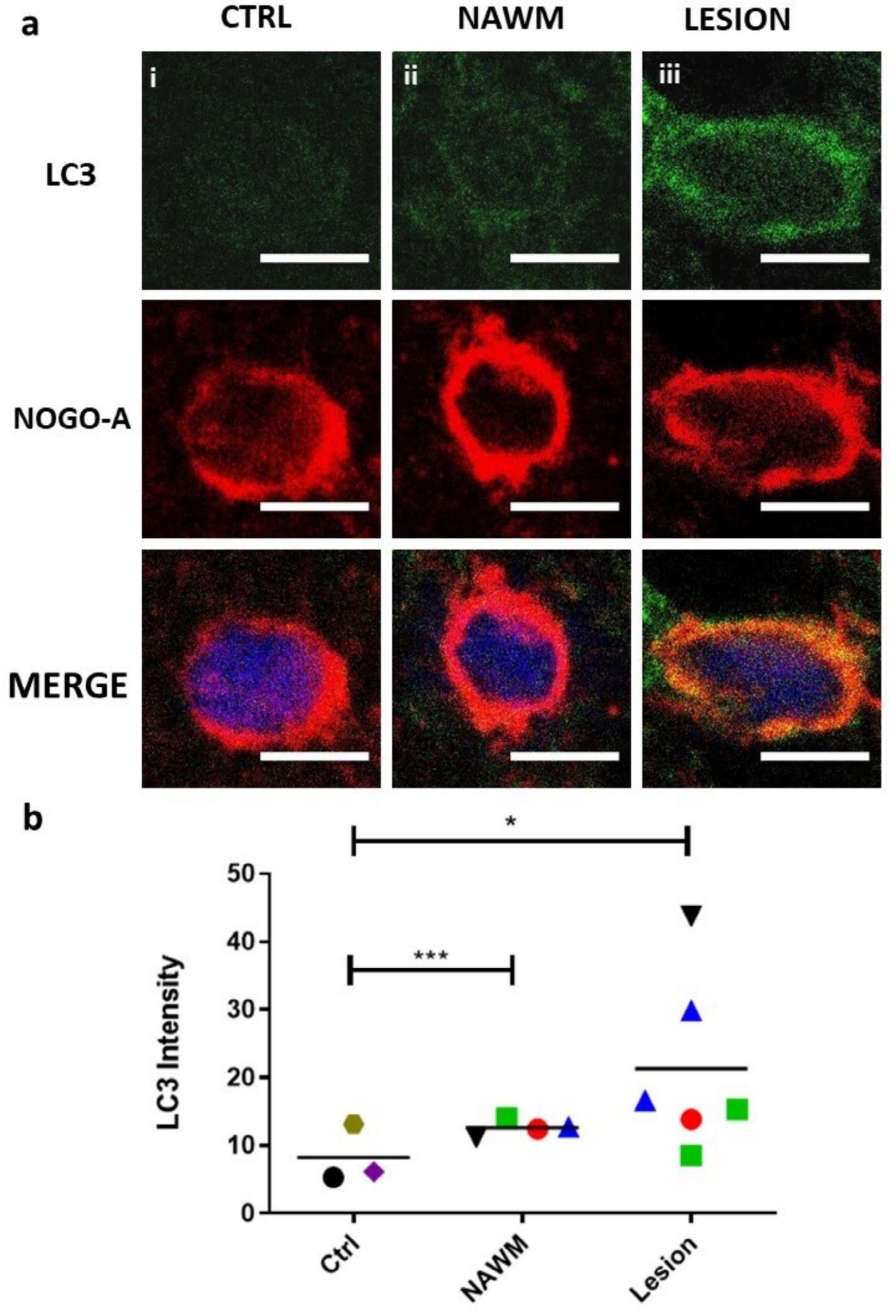
Increased expression of the autophagy marker LC3 in MS lesions and NAWM compared to controls. **a** Sample images showing relative expression of LC3 in a non-MS CTRL case, in NAWM from an MS case, and in a chronic active lesion of an MS case. Scale bars correspond to 5 μm. **b** Quantification of LC3 expression as measured by average immunofluorescence intensity of LC3 in OLs in healthy controls, NAWM, and chronic active MS lesions. Individual regions of interest are indicated by color and shape corresponding to 3 non-MS controls, 4 NAWM regions, and 6 lesions from 4 individuals with MS. Statistical significance was assessed using Student’s t-test: * (< 0.05), *** (< 0.001)

*Increased presence of autophagosomes in OLs *in situ* in MS* – To determine whether autophagosomes accumulate in situ in cases of MS, akin to their accumulation in hOLs in response to metabolic stress in vitro. we co-labeled tissue sections containing chronic active lesions with antibodies for NOGO A for OLs and LC3 as a marker of autophagosomes. Examples of the range of LC3 expression are provided in Fig. 2a. Expression of LC3 in OLs was significantly increased in the MS cases, both in the chronic active lesions and in normal appearing white matter (NAWM), compared to control tissue samples from non-MS cases (Fig. 2b).

**Fig. 3:**
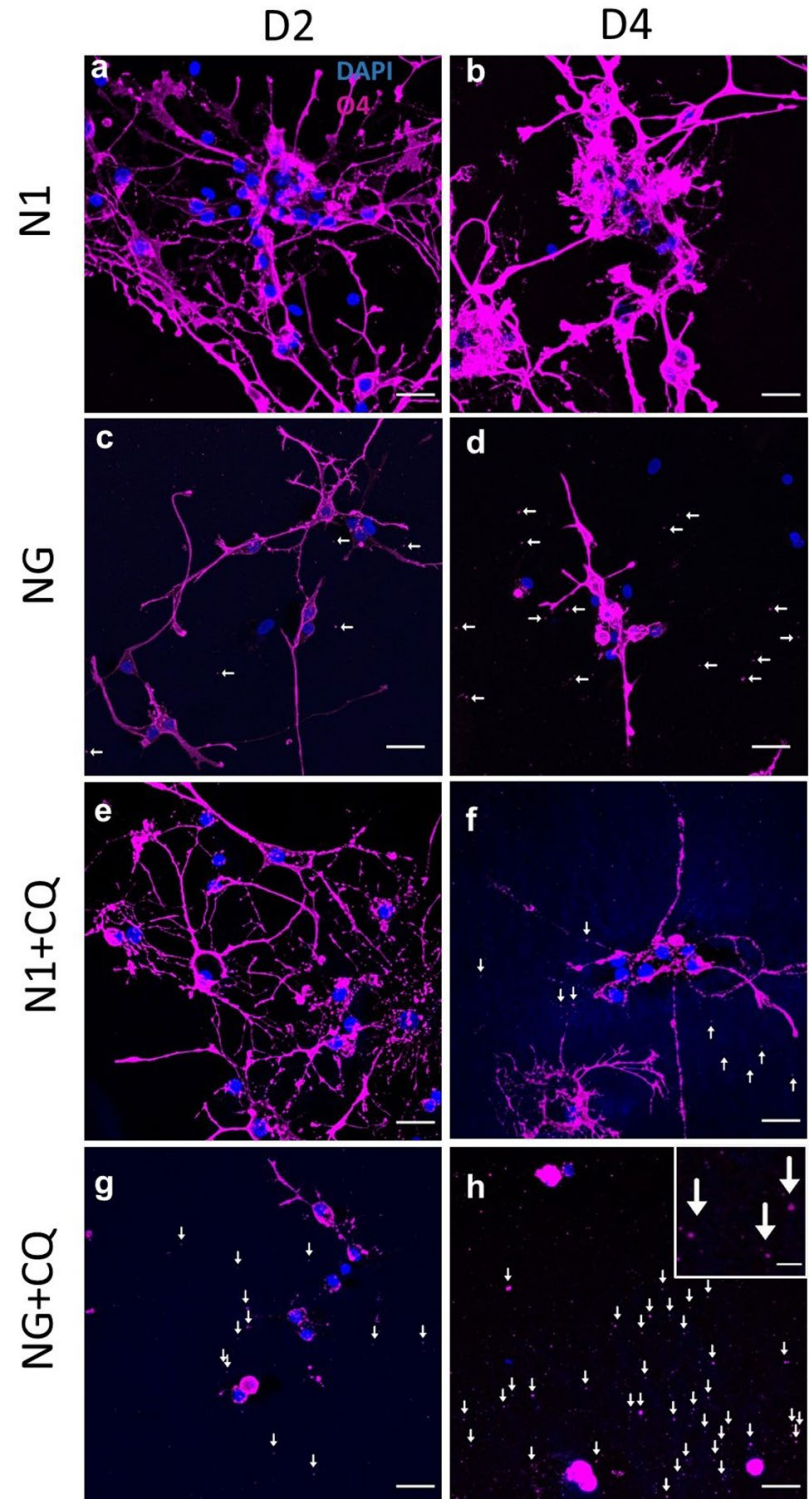
No glucose conditions and treatment with chloroquine cause cell process loss and shedding of membrane fragments. **a, b** hOLs project long processes in N1 conditions at 2 and 4 days in vitro (DIV). **c, d** With NG, processes show signs of contraction. O4 positive fragments are visible in the media (indicated by arrows). **e** In N1 conditions combined with chloroquine, the morphology of hOLs was similar to N1 conditions without chloroquine after 2 DIV. **f** After 4 days of treatment in N1 combined with chloroquine, some process retraction and some O4 + fragments can be observed outside the cell (fragments indicated by arrows). **g, h** Combined treatment of NG and chloroquine after 2 and 4 DIV resulted in greater retraction of processes and the presence of many fragments outside the cell. Cell size was decreased. The inset picture in **h** illustrates a magnified view of the O4 positive fragments. Scale bars correspond to 20 μm in the large figures and 2.5 μm in the inset in panel **h**

*Autophagy inhibition causes cell process loss and shedding of membrane fragments -* We have previously observed hOL process retraction at day 6 when challenged by metabolic stress alone [37]. Comparing N1 conditions alone (Fig. 3a-b), NG conditions alone (Fig. 3c-d), or N1 conditions containing the autophagy inhibitor chloroquine (Fig. 3e-f), hOLs treated with chloroquine under NG conditions exhibited a marked reduction of cellular process thickness and length by 2 and 4 days (Fig. 3g-h). NG conditions or N1 conditions combined with chloroquine, resulted in a limited number of small O4-positive membranous fragments outside the cells (Fig. 3c-f). These were not observed in N1 conditions alone (Fig. 3a-b). Inhibition of autophagy with chloroquine in NG conditions resulted in a larger number of extracellular O4-positive fragments (Fig. 3g-h).

### hOL loss and shrinkage of nuclear size in MS lesions and under metabolic stress in vitro

In a previous study, we evaluated OL cell numbers in MS lesions characterized as active/demyelinating, active post-demyelinating, and mixed active/inactive lesions. [20] For active demyelinating lesions, characterized by the presence of macrophages/microglia throughout the whole lesion area and a significant subset of these phagocytes containing myelin degradation products [25], we observed OL numbers comparable to normal appearing white matter (NAWM) [20]. In contrast, mixed active/inactive lesions, which have a hypocellular lesion center and a rim of macrophages/microglia at the lesion border (subsequently named mixed lesions) as well as inactive lesions, which are almost completely devoid of phagocytes, show an almost complete loss of oligodendrocytes [20]. In active post-demyelinating lesions, which represent a transition stage from active/demyelinating lesions to either mixed lesions or inactive lesions, macrophages/microglia are present throughout the lesion areas, but there are no myelin degradation products within the phagocytes. OL numbers are variably reduced in this lesion type, as are the mean numbers of CD68 positive macrophages/microglia compared to active/demyelinating lesions [20]. For the current study, we selected 9 “active/post-demyelinating” lesions from autopsies from 4 patients. All lesions were in the white matter of the brain (*n* = 8) or the cerebellum (*n* = 1). An example of such a lesion is provided in Supplementary Fig. 2. We confirmed our previous findings (Hess et al. [20] of reduced numbers of OLs (178 +/- 79 cells/mm^2^ vs. 1081 +/- 27 cells/mm^2^) and macrophages/microglia (715 +/- 111 /mm^2^ vs. 2491 +/- 112 mm^2^) in active/post-demyelinating lesions versus active/demyelinating lesions. We observed a high variability in the number of macrophages/microglia and oligodendrocytes between the individual lesions (between 2 and 618 OLs/mm^2^ and between 283 and 1333 macrophages/microglia/mm^2^). In lesions with a relative preservation of OLs, we found a graded loss of OLs from the lesion border to the lesion center (Supplementary Fig. 2d).

**Fig. 4:**
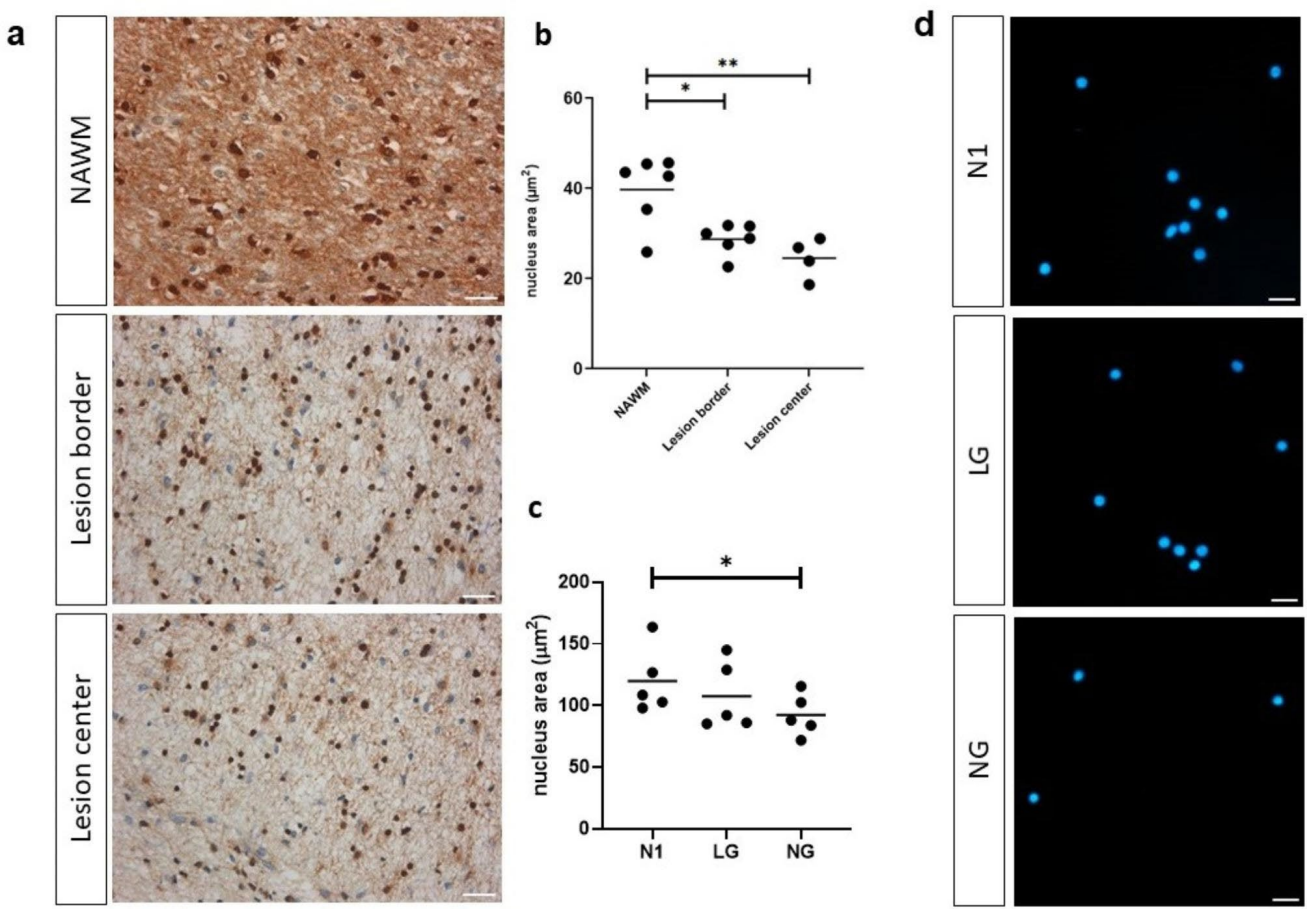
Shrinkage of hOL nuclear size in MS lesions and under metabolic stress in vitro. **a** Sample images of an MS case showing nucleus size in NAWM, lesion edge, and lesion center. Scale bars correspond to 10 μm. **b** Quantification of nuclear size (surface area) in NAWM, lesion edge, and lesion center, showing nuclear size reduced in the lesion center and edge compared to NAWM. Statistical significance was assessed using ANOVA/Dunnett’s test: *(< 0.05), **(< 0.01). **c** Quantification of nuclear size in optimal (N1), low glucose (LG) and no glucose (NG) conditions at 4 days, showing nuclear size reduced under metabolic stress. Statistical significance was assessed using ANOVA/Dunnett’s test: *(< 0.05). **d** Sample images illustrating nucleus size (DAPI staining) in vitro under N1, LG and NG conditions. Scale bars correspond to 20 μm

Nuclear condensation and volume reduction (pyknosis) are hallmarks of cell death. Pyknosis can occur in two forms: nucleolytic and anucleolytic. Metabolic stress causes anucleolytic pyknosis [5]. We observed a significant decrease in nuclear area size of surviving OLs within the lesions compared to OLs in NAWM (Fig. 4a-b). Under in vitro stress conditions, mean nuclear size was reduced in OLs at day 4 (Fig. 4c-d), a time when significant cell death was initially detected [6].

### Limited contribution of ROS to hOL injury under metabolic stress

**Fig. 5:**
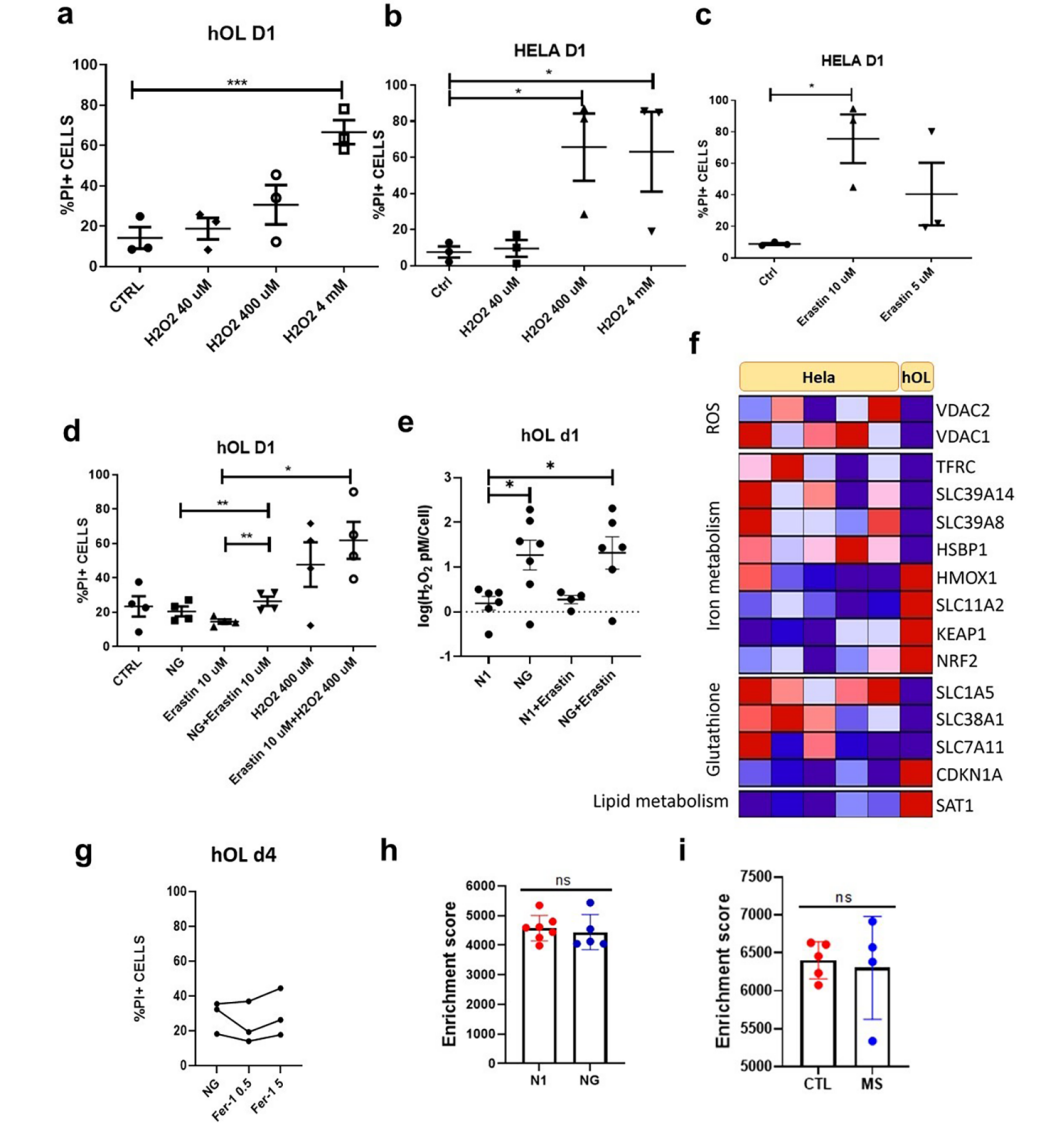
ROS mediates limited damage in hOL following metabolic stress. **a-b** hOLs and HeLa cells were treated with hydrogen peroxide for 1 day at different concentrations and rate of cell death was measured by PI assay. **a** hOL cell death was increased only by 4 mM H_2_O_2_. **b** HeLa cell death was substantially increased by 400 μm of H_2_O_2_. Statistical significance was assessed using an ANOVA/Tukey test: *(< 0.05), ***(< 0.001). **c** HeLa cells were treated with Erastin at different concentrations for 1 day. Cell death was significantly increased by treatment with 10 µM of erastin. Statistical significance was assessed using a Student’s t-test: *(< 0.05). **d** hOLs were treated with Erastin, NG, H_2_O_2_ or combination of these treatments for 1 day. Cell death was only significantly increased with the combined treatment of Erastin with NG and not with NG or Erastin alone. Statistical significance was assessed using a Student’s t-test: *(< 0.05), **(< 0.01). **e** H_2_O_2_ levels measured in hOLs treated with Erastin in optimal and NG conditions. H_2_O_2_ levels were increased under NG conditions alone. No additional effect of Erastin was detected. Statistical significance was assessed using an ANOVA/Dunnett’s test: *(< 0.05). **f** Genes presenting significant difference in their transcriptional expression in hOLs compared to HeLa cells were categorised according to the pathway involved in ferroptosis. Genes upregulated are shown in red and downregulated in blue. HeLa cell data were obtained from publicly available databases [1, 10, 17]. hOLs data was obtained from a bulk RNA sequencing database that we have previously published [31]. **g** Rate of hOL cell death under NG conditions following treatment with Ferrostatin-1 for 4 days, measured by PI assay. No significant differences were observed between treatment conditions. Each dot/line in the graphs corresponds to an independent biological sample. Statistical significance was assessed using an ANOVA/Dunnett’s test. **h** Comparison of the enrichment score of genes related to ferroptosis in N1 and NG conditions in vitro. Mean ± SEM for each condition shown in the figure. Statistical significance was assessed using a Student’s t-test: *(< 0.05). **i** Enrichment score of genes related to ferroptosis in MS cases compared to “control” individuals. Mean ± SEM for each condition shown in the figure. Statistical significance was assessed using a Student’s t-test: *(< 0.05)

We compared the susceptibility of primary hOLs to exogenous oxidative stress to HeLa cells, a cell type used in previous studies of this mechanism of injury. [16]. Addition of H_2_O_2_ as an exogenous source of ROS is toxic to hOLs only at concentrations greater than required for HeLa cells, i.e., the proportion of PI-positive cells following treatment with 400 µM of H_2_O_2_ is higher in HeLa cells than hOLs (Fig. 5a-b). As glutathione protects against ROS, we exposed hOLs and HeLa cells to Erastin, which inhibits glutathione synthesis. Erastin alone applied for 24 h, as previously shown [16], results in significant HeLa cell death (Fig. 5c). In contrast, primary hOLs under N1 conditions were resistant to Erastin induced cell death (Fig. 5d). Under NG conditions, Erastin increased hOL cell death (Fig. 5d), and was associated with increased ROS within 1 day (Fig. 5e). These data indicate that anti-oxidative mechanisms are limiting the injury effects of the increased ROS produced in the hOLs by metabolic stress conditions.

To identify a mechanistic basis for hOL resistance, we compared expression of ROS and iron regulating genes between hOLs and HeLa cells cultured under basal conditions. Examining ROS related pathways, we found that VDAC1 and VDAC2, which control the exchange of small metabolites between the mitochondria and the cytosol [27], are relatively downregulated in the hOLs, potentially contributing to reduction of ROS formation (Fig. 5f). Examining gene products that contribute to the regulation of iron levels, we detected increased expression of HMOX1, which degrades the heme molecular complex, along with its regulators NRF2 and KEAP1 [27]. Genes related to iron uptake, SLC39A14, SLC39A8, SLC11A2, TRFC and a transcription factor that regulates their expression, HSPB (with the exception of SLC11A2 [27]) were downregulated in hOLs as a result of metabolic stress. In contrast, the iron export-related gene SLC40A1 [27] was upregulated. Three genes involved in glutathione precursors uptake, SLC1A5, SLC38A1 and SLC7A11, are downregulated, while CDKN1A, a gene responsible for reduction in sensitivity to ferroptosis, is upregulated [27]. Amongst four key gene products related to lipid metabolism in the regulation of ferroptosis [27], only SAT was differentially regulated. These transcriptional profiles suggest that hOLs are more resistant to iron accumulation than HeLa cells.

*Lack of evidence of Ferroptosis* - endogenous oxidative stress is shown to react with iron to cause lipid peroxidation. This can trigger the RCD referred to as ferroptosis. To determine if these treatments triggered mechanisms associated with ferroptosis mediated cell death, we applied ferrostatin-1. No protection from cell death was detected (Fig. 5g). We have also used buthionine sulfoximine (BSO) to evaluate ferroptosis inhibition; this agent also had no effect on hOL cell death induced by metabolic stress (data not shown).

Using an enrichment score of pathways mediating a wider range of RCD pathways, we did not observe any differences in the ferroptosis related transcriptome of primary hOLs cultured under optimal versus metabolic stress conditions (Fig. 5h). Using nuclear RNA sequencing databases from MS tissues [1, 10, 17], this RCD pathway was not activated in OLs in the MS cases compared to control tissues (Fig. 5i).

### Calcium-dependent mechanisms degrade hOL cytoskeleton components under metabolic stress

**Fig. 6:**
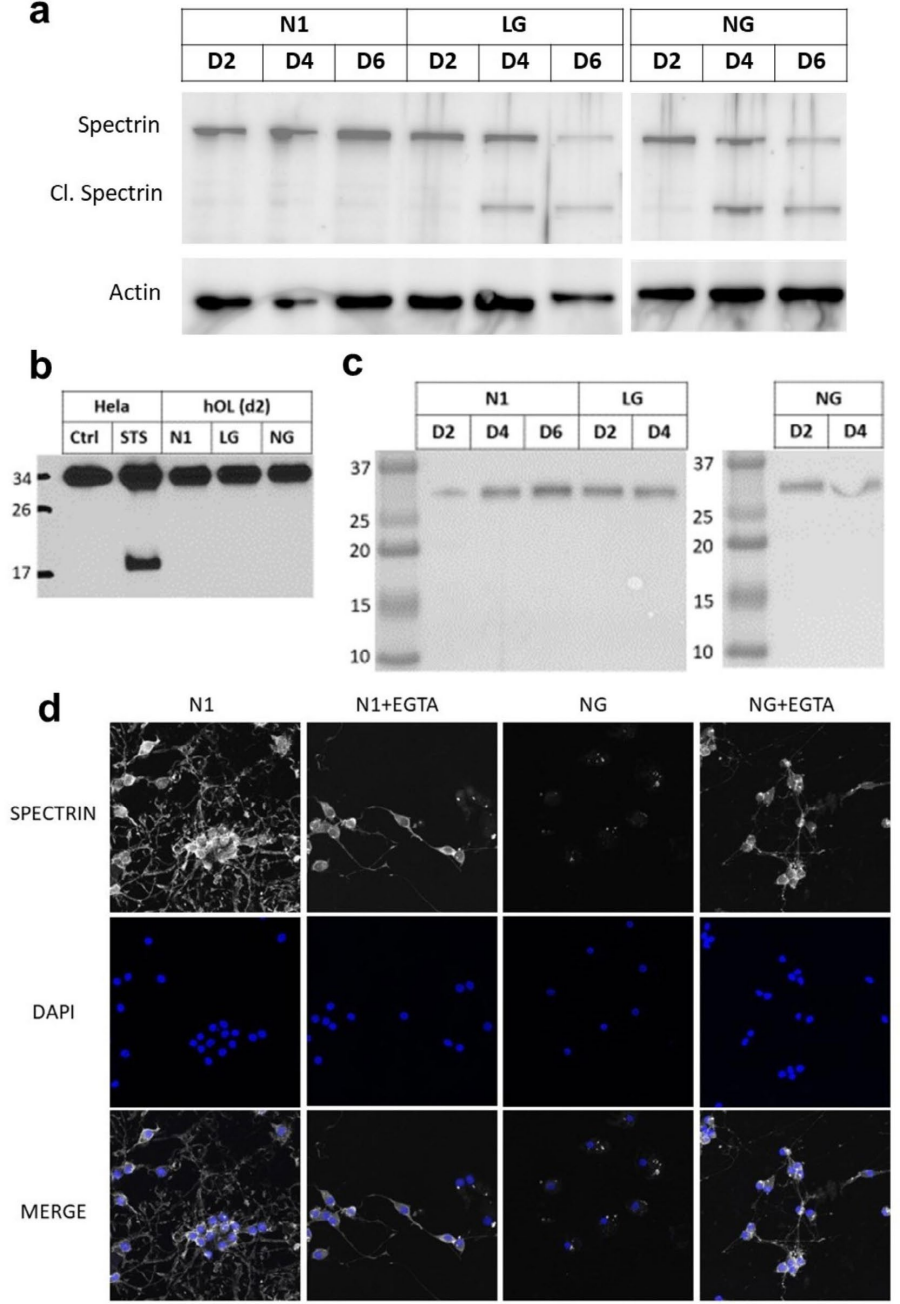
hOL cytoskeleton is degraded by a mechanism dependent on calcium activation. **a** hOLs were cultured in N1, LG and NG conditions for 2, 4 and 6 days. Spectrin cleavage was assessed by Western Blot. **b** Hela cells were treated with the apoptosis activator staurosporine and hOL were cultured in N1, LG and NG conditions for 2 days. Procaspase-3 is detected at 32 kDa, while cleaved caspase-3 at 17 kDa. **c** In the same sample used in a), cleavage of caspase-3 was not detected after 4 days in low and no glucose conditions. **d** hOLs were treated under N1 and NG conditions in combination with EGTA for 4 days and immunocytochemistry for Spectrin and O4 (hOL marker) was performed, followed by confocal imaging.

We hypothesized that decreased intracellular ATP, due to metabolic stress, could lead to an increase in cytosolic Ca^2+^ concentration [Ca^2+^]_i_ and that this could contribute to hOL degeneration via activation of calcium-dependent proteases that target the cytoskeleton [32]. We therefore evaluated cleavage of spectrin, a substrate of the calcium-dependent protease calpain. Cleavage of spectrin was detected after 2 and 4 days in low glucose and no glucose conditions (Fig. 6a). As spectrin is also a substrate of caspase-3, we performed western blots using an anti-caspase-3 antibody capable of detecting both procaspase-3 and its activated cleaved form. Cleaveage of caspase-3 was not identified after 2 days of treatment, either in low or no glucose conditions, in contrast to Hela cells treated with staurosporine (Fig. 6b). Using the same sample as in Fig. 6a, we also did not detect cleavage of caspase-3 after day 4 when cultured in low and no glucose conditions (Fig. 6c).

To further test the hypothesis that activation of calcium-dependent proteases is critical to the hOL cell death induced by metabolic stress, the cells were treated with the calcium chelator EGTA in combination with the no glucose condition. At day 4, we determined that spectrin is preserved in hOLs in EGTA treated no glucose cultures, in contrast with the no glucose alone condition, in which spectrin had been almost completly degraded (Fig. 6d).

These findings support the hypothesis that the activation of a calcium-dependent protease contributes to the metabolic stress induced degradation of the hOL cytoskeleton.

**Fig. 7:**
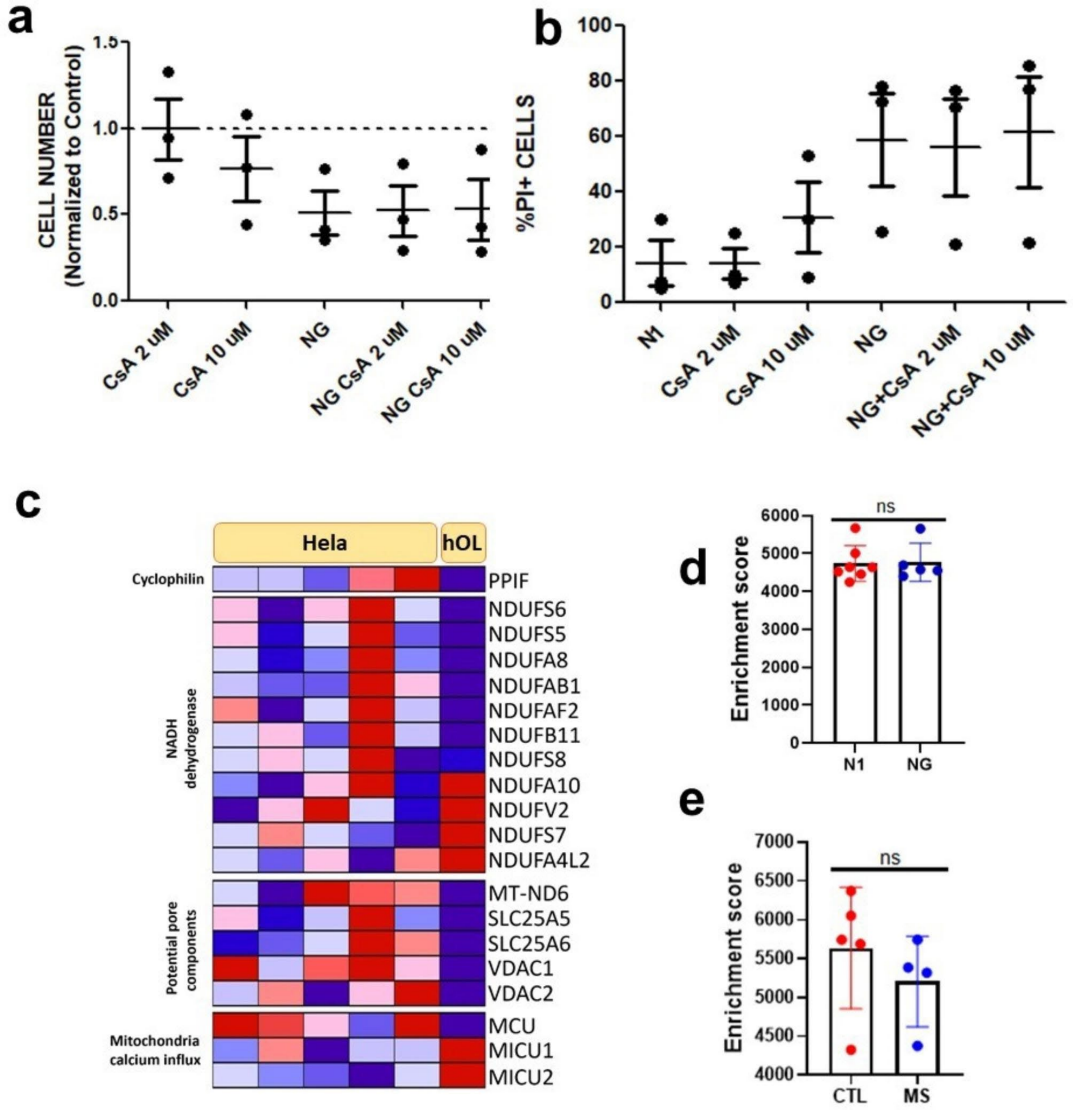
Metabolic stress triggers spectrin cleavage without activation of MPT-driven necrosis. **a-b** hOLs treated with CsA at 2 µM and 10 µM in combination with N1 and NG conditions did not cause a reduction in cell number or increase in PI-positive cells after 4 days of treatment. Each dot in the graphs corresponds to an independent biological sample. Mean ± SEM for each condition is shown in the figure. Statistical significance was assessed using a ANOVA/Dunnett’s test. **c** Genes presenting significant differences in their transcriptional expression in hOLs compared to HeLa cells were categorised according to pathways involved in MPT-driven necrosis. Genes upregulated are shown in red and downregulated in blue. HeLa cell data was obtained from publicly available databases [1, 10, 17]. hOLs data was obtained from a bulk RNA sequencing database that we have previously published [31]. **d** Comparison of the enrichment score of genes related to MPT-driven necrosis in N1 and NG conditions in vitro. Mean ± SEM for each condition shown in the figure. Statistical significance was assessed using a Student’s t-test: *(< 0.05). **e** Enrichment score of genes related to MPT-driven necrosis in MS cases compared to “control” individuals. Mean ± SEM for each condition shown in the figure. Statistical significance was assessed using a Student’s t-test: *(< 0.05)

*Lack of evidence of MPT mediated necrosis* - This RCD pathway triggers cell death by opening the mitochondrial permeability transition pore complex due to overloaded cytosolic calcium [12]. We determined if the increased cytoplasmic calcium described above might be linked to MPT-driven necrosis. To test this, primary hOLs were treated with cyclosporin A, which inhibits cyclophilin, a calcium activated protein that regulates the transition pore complex. [2] No changes were detected in cell number or cell death compared to untreated cells in N1 or NG conditions (Fig. 7a-b).

Examining the expression of MPT related genes, PPIF, the gene encoding cyclophilin, is expressed at a lower level in hOLs compared to HeLa cells (Fig. 7c). No consistent differences in gene expression were detected between hOLs and HeLa cells for gene products related to calcium influx (Fig. 7c). Among MPT pore related genes, we identified five that are expressed at a significantly lower level in hOLs than HeLa cells (Fig. 7c). Overall, the transcriptomic analysis of genes encoding proteins that regulate MPT pore function suggest that hOLs are more resistant to MPT pore formation than HeLa cells. As with our ferroptosis enrichment score analyses, we did not observe any differences in the transcriptomes of primary hOLs cultured under optimal versus metabolic stressed conditions (Fig. 7d) or any significant upregulation of this pathway in OLs from MS lesions (Fig. 7e).

Our findings provide evidence that metabolic stress results in elevated intracellular calcium concentration [Ca^2+^]_i_ in hOLs, yet without activation of MPT driven necrosis.

## Discussion

Here we investigate the mechanisms underlying the distinct injury response of human mature OLs to metabolic stress.

### Metabolic stress reduces cellular ATP and compromises autophagic flux in hOLs

ATP depletion - Our time-course studies showed that the delayed cell death of hOLs was preceded by a significant and progressive reduction in ATP per cell. We previously used a Seahorse bio-analyzer to show that hOLs when challenged with metabolic stress exhibit a strong dependence on glycolysis and a comparatively low rate of ATP production [42]. When internal levels of ATP are low, AMP-activated protein kinase (AMPK) is activated. This kinase inhibits the activity of the mammalian target of rapamycin (mTOR), causing a reduction in protein synthesis and activation of autophagy [13, 14, 15, 35]. Autophagy provides substrates for the tricarboxylic acid (TCA) cycle [15]. However, as mentioned, hOLs exhibit a reduced reliance on oxidative phosphorylation and are more dependent on glycolysis for energy production [42]. We did not observe any significant effect of inhibition or activation of autophagy with chloroquine or Torin-1 on ATP levels under either basal or stress conditions, suggesting that in hOLs autophagy is not a major mechanism contributing to the modulation of energy production.

Autophagy failure – we used LC3, a marker of autophagosomes to assess autophagy status in vitro (in combination with LAMP1) and in MS cases. Our data indicate that autophagy failure occurs under sustained metabolic stress. As sustained activation of autophagy depends on ATP [46], this may be a consequence of ATP depletion. Autophagosomes are transported by dyneins while lysosomes are transported by kinesins. Both of these families of motor molecules depend on ATP for motility [50]. The fusion between autophagosomes and lysosomes depends on RAB7, a small GTPase, also dependent on ATP availability. Thus a lack of ATP would impair the transport and fusion of autophagosomes and lysosomes and cause the autophagy failure observed in hOL under metabolic stress [50]. We confirmed our previous observation that inhibiting autophagy progression exaggerates cell death [9].

Assessing autophagosome formation and fusion with lysosomes in vitro, after 2 days of metabolic stress when ATP levels were already reduced, we found limited buildup of unfused autophagosomes, indicating a functioning if not heightened autophagy pathway activation. Autophagy, however, was not able to sustain ATP levels and we did not detect a burst of ATP production at the earliest time points tested under stress conditions. With prolonged stress, accumulation of unfused autophagosomes was present, indicating autophagy failure. Such failure can lead to a disruption in the regulation of cytoskeleton remodeling as identified by He el al. [18] or an impairment of myelination as shown by Karim et al. [24], We detected many O4-positive fragments outside the cells indicating that cellular material is being lost to the environment.

Our in situ studies of MS cases parallel our in vitro findings. We detected significant reduction of OL numbers in both our in vitro model and active post demyelinating and chronic active lesions [20]. We now document reduced size of nuclei (pyknosis) [5] in hOLs in vitro and in MS lesions, suggesting substantial cellular distress and potentially ongoing cell death. Immunohistochemical analyses of MS cases revealed a build-up of autophagosomes (increased expression of LC3) in OLs serving as an indicator of metabolic stress. We note that Satoh et al. did not detect LC3 expression in OLs in MS cases, in comparison to the high expression noted in cases of Nasu-Hakola disease [43]. The increase of LC3 in NAWM indicates that stress is not restricted to the lesion area, potentially preceeding the formation of new lesions.

### Basis of metabolic stress induced cell injury

Limited contribution of ROS to hOL injury **-** The stress conditions we applied to hOLs in vitro increased the production of ROS, which promotes lipid peroxidation. Further, combining Erastin with glucose deprivation increased cell death, indicating a protective role for antioxidants. Our functional data however suggest that ROS makes a relatively minor contribution to hOL injury induced by these metabolic stress conditions, possibly because hOLs have a lower rate of oxidative phosphorylation compared to rat OLs and OPCs [42].

Our transcriptomic analyses provide insight into the mechanism underlying the relative resistance of hOLs to exogenous H_2_O_2_ compared to HeLa cells. Our findings revealed downregulation of VDAC1 and VDAC2, that control the exchange of small metabolites between the mitochondria and the cytosol [27], as well as resistance to iron accumulation in hOLs compared to HeLa cells. These differences in transcriptional profile could compensate for the observed reduction of glutathione synthesis related genes. We then addressed if the production of ROS under stress conditions triggered ferroptosis [12, 23, 33, 34]. Blocking ferroptosis with ferrostatin did not protect the cells from death under the stress conditions applied here, supporting the conclusion that ferroptosis is not the primary underlying mechanism of cell death. Analyses of publicly available molecular databases did not identify changes in expression of the ferroptosis RCD pathway in OLs in active MS lesions [22].

Increase in [Ca^2+^]_i_ and activation of Ca^2+^-dependent proteases - Our findings support the hypothesis that metabolic stress triggers hOL cell death via a mechanism that depends on an increase in [Ca^2+^]_i_ that then leads to the proteolytic degradation of the cytoskeleton. Myelin produced by hOLs is a plastic structure that constantly adjusts its morphology to adapt to neural activity. Local changes in myelin sheets are dependent on Ca^2+^ signaling that is regulated by voltage-gated and ligant-gated channels, modulating conformation changes in the cytoskeleton via Ca^2+^ influx [36]. Considerable energy is required to maintaint the calcium gradient across the plasma membrane, engaging the plasma membrane calcium ATPase transporter (PMCA) [11]. Calcium is also extruded by the Na^+^/Ca^2+^-exchanger (NCX) and Na^+^/Ca^2+^/K^+^-exchanger (NCKX) [11]. Low levels of ATP in the cell compromizes the capacity of the cell to maintain the steep ionic gradients across the plasma membrane, which can ultimately lead to metabolic collapse and cell death.

As a readout of abnormally high cytoplasmic calcium, we documented metabolic stress induced cleavage of spectrin, a major cytoskeletal component that is a target of the Ca^2+^-dependent protease calpain [39]. This cleavage was not caspase-3 dependent but was inhibited by chelating extracellular Ca^2+^ with EGTA. Possible contributing sources of the increased intracellular Ca^2+^ include transmembrane flux from the extracellular space, or release from intracellular stores including endoplasmic reticulum and mitochondria [2, 49]. Notably, release of calcium from intracellular stores can activate the MPT-driven necrosis RCD pathway [12], however, addition of cyclosporine A to inhibit cyclophilin, a central participant in mitochondrial pore opening [48], did not protect hOLs in the conditions tested, providing evidence that MPT-driven necrosis does not contribute to hOL death in these conditions.

## Conclusion

**Fig. 8:**
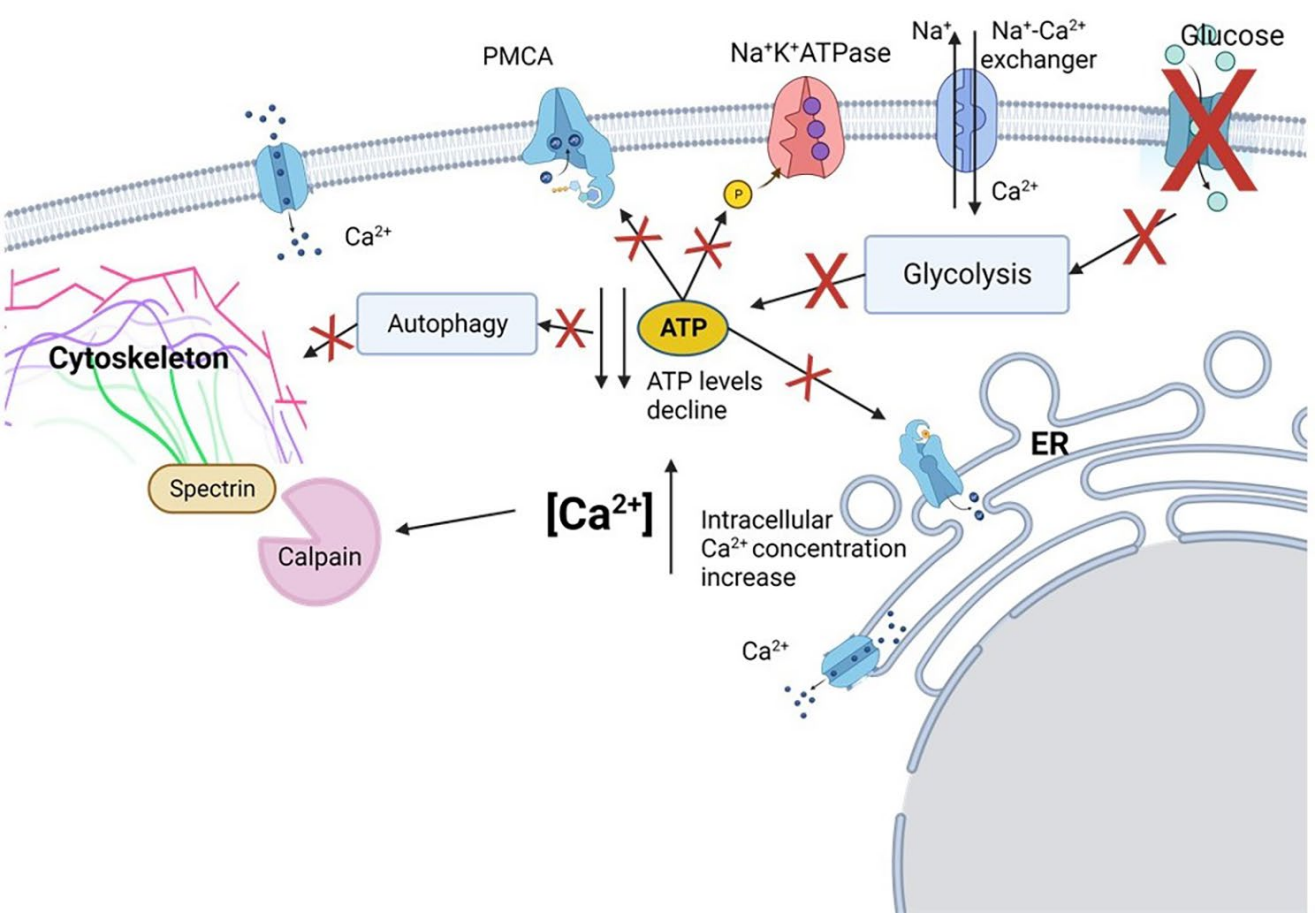
Mechanisms of human OL lethal injury. In the absence of glucose uptake, glycolysis, the main source of ATP in hOL, is reduced. As ATP is necessary for the transport and fusion of autophagosomes and lysosomes, autophagy is impaired. Autophagy blockage results in process disruption in OLs, indicating that this mechanism is important for the structural integrity of the cell. Reduction in ATP levels also causes a decrease in the activity of ATP-dependent Ca^2+^ pumps, like plasma membrane calcium ATPase (PMCA), and the Na^+^K^+^ATPase, leading to an increase in cytosolic Ca^2+^ and Na^+^. The raise in intracellular Na^+^ increases the activity of Na^+^-Ca^2+^ exchangers, what causes an additional influx of Ca^2+^. High levels of Ca^2+^ activate Ca^2+^-dependent proteases as calpain, which cleave spectrin, an essential component for the integrity of the cytoskeleton. These mechanisms initially contribute to loss of hOL processes (sub-lethal injury) and subsequently to cell death. Figure created using Biorender.

Here we demonstrate that the response of hOLs to metabolic stress is distinct from RCD mechanisms that are readily triggered in other cell types (Fig. 8). Although the cause of OL cell death in MS cases has not yet been identified, our studies of hOLs in vitro indicate that metabolic failure is the most consistent cause of death [6, 9, 37, 42]. Other mediators of cellular stress, particularly cytokines, resulted in only sublethal injury without significant cell death [37]. We consider that the hOL cell death described here results primarily from energy depletion, followed by autophagy failure. Our studies link the distinct response of these cells to metabolic insults to their low basal metabolic state and dependence on glycolytic metabolism [40, 42], together with a genetic program of resistance to RCD activation. Notably, our detection in vitro of cell and nuclear shrinkage, autophagosome accumulation, and lack of RCD activation is paralleled by in situ observations of MS lesion. We postulate that the observed release of cellular contents would engage interactions with surrounding glia, engaging an ongoing injury response. Defining the distinct bases of OL injury and death provides guidance for the development of neuro-protective interventions.

## Electronic supplementary material

Below is the link to the electronic supplementary material.

**Supplementary Material 1**. **Supplementary Table 1**: Clinical details of samples used for functional and biochemical assays; **Supplementary Figure 1**: Metabolic stress induces activation of AMPK in hOL; **Supplementary Figure 2**: Loss of hOL is intensified in the center of MS lesions

## Data Availability

The dataset used and/or analysed during the current study are available from the corresponding author on reasonable request.

## List of abbreviations

OL: Oligodendrocyte
hOL: Human oligodendrocyte
RCD: Regulated cell death
MS: Multiple sclerosis
ISR: Integrated stress response
MPTN: Mitochondrial permeability transition-driven necrosis
CRCHUM: Centre Hospitalier de l’Université de Montréal
LFB: Luxol Fast Blye
H&E: Hematoxylin and Eosin
MNI: Montreal Neurological Institute
LG: Low glucose
NG: No glucose
PI: Propidium iodide
ssGSEA: Single samples gene set enrichment analysis
GEO: Gene expression omnibus
N1: Optimal media
CQ: Chloroquine
NAWM: Normal appearing white matter
DIV: Days in vitro
BSO: Buthionine sulfoximine
AMPK: AMP-activated protein kinase
mTOR: Mammalian target of rapamycin
TCA: Tricarboxylic acid
PMCA: Plasma membrane calcium ATPase transporter
NCX: Na^+^/Ca^2+^-exchanger
NCKX: Na^+^/Ca^2+^/K^+^-exchanger
