# Supplementary material for: Mechanisms of metabolic stress induced cell death of human oligodendrocytes: relevance for progressive multiple sclerosis
Source: Acta Neuropathol Commun. 2023 Jul 5;11:108. doi: 10.1186/s40478-023-01601-1 (PMC10320974; doi:10.1186/s40478-023-01601-1)
Supplement: Supplementary file 1 — Supplementary Material 1. Supplementary Table 1: Clinical details of samples used for functional and biochemical assays; Supplementary Figure 1: Metabolic stress induces activation of AMPK in hOL; Supplementary Figure 2: Loss of hOL is intensified in the center of MS lesions [file 40478_2023_1601_MOESM1_ESM.docx]

**Supplementary Information**

**Supplementary Table 1. Clinical details of samples used for functional and biochemical assays**

| Prep | Age | Sex | ATP 6h | ATP 2/4 d | Autophagy | Nucleus | H_2_O_2_/PI | Erastin/PI | H_2_O_2_ | Fer-1 | WB | CsA | IHC |
| --- | --- | --- | --- | --- | --- | --- | --- | --- | --- | --- | --- | --- | --- |
| HA820 | 12 | F |  |  |  | + |  |  |  |  |  |  |  |
| HA831 | 16 | F |  |  |  | + |  |  |  |  |  |  |  |
| HA832 | 11 | M |  |  |  | + |  |  |  |  |  |  |  |
| HA833 | 11 | M |  |  |  | + |  |  |  |  |  |  |  |
| HA834 | 23 | F |  |  |  | + |  |  |  |  |  |  |  |
| HA836 | 42 | M |  | + |  |  |  |  |  |  |  |  |  |
| HA837 | 28 | F |  | + |  |  |  |  |  |  |  |  |  |
| HA838 | 13 | F |  | + |  |  |  |  |  |  |  |  |  |
| HA841 | 24 | M |  | + |  |  |  |  |  |  |  |  |  |
| HA842 | 68 | M |  | + | + |  |  |  |  |  |  |  |  |
| HA843 | 8 | F |  | + | + |  |  |  |  |  |  |  |  |
| HA849 | 54 | M |  | + |  |  | + |  |  |  |  |  |  |
| HA850 | 64 | F |  | + |  |  | + | + |  |  |  |  |  |
| HA851 | 18 | F |  | + | + |  | + | + |  |  |  |  |  |
| HA853 | 6 | N |  |  |  |  |  | + |  |  |  |  |  |
| HA854 | 39 | F |  |  |  |  |  | + |  |  |  |  |  |
| HA855 | 48 | F |  |  |  |  |  |  |  |  |  | + |  |
| HA857 | 13 | M |  |  |  |  |  |  |  |  |  | + |  |
| HA858 | 12 | F |  |  |  |  |  |  | + |  |  | + |  |
| HA859 | 44 | F |  |  |  |  |  |  | + |  | + |  |  |
| HA861 | 14 | F |  |  |  |  |  |  | + |  |  |  |  |
| HA862 | 26 | M | + |  |  |  |  |  |  |  |  |  |  |
| HA863 | 32 | F | + |  |  |  |  |  |  |  |  |  |  |
| HA865 | 56 | M | + |  |  |  |  |  |  |  |  |  |  |
| HA867 | 12 | F |  |  |  |  |  |  |  |  |  |  |  |
| HA868 | 75 | M |  |  |  |  |  |  |  |  |  |  |  |
| HA869 | 10 | M |  |  |  |  |  |  |  |  |  |  |  |
| AB103 | 48 | M |  |  |  |  |  |  |  |  |  |  |  |
| AB129 | 33 | F |  |  |  |  |  |  |  |  |  |  | + |
| AB187 | 26 | M |  |  |  |  |  |  |  |  |  |  | + |
| AB203 | 44 | F |  |  |  |  |  |  |  |  |  |  | + |
| AB159 | 67 | M |  |  |  |  |  |  |  |  |  |  | + |
| P18 | 59 |  |  |  |  |  |  |  |  |  |  |  | + |
| P24 | 56 |  |  |  |  |  |  |  |  |  |  |  | + |
| P30 | 61 |  |  |  |  |  |  |  |  |  |  |  | + |


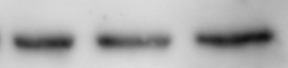

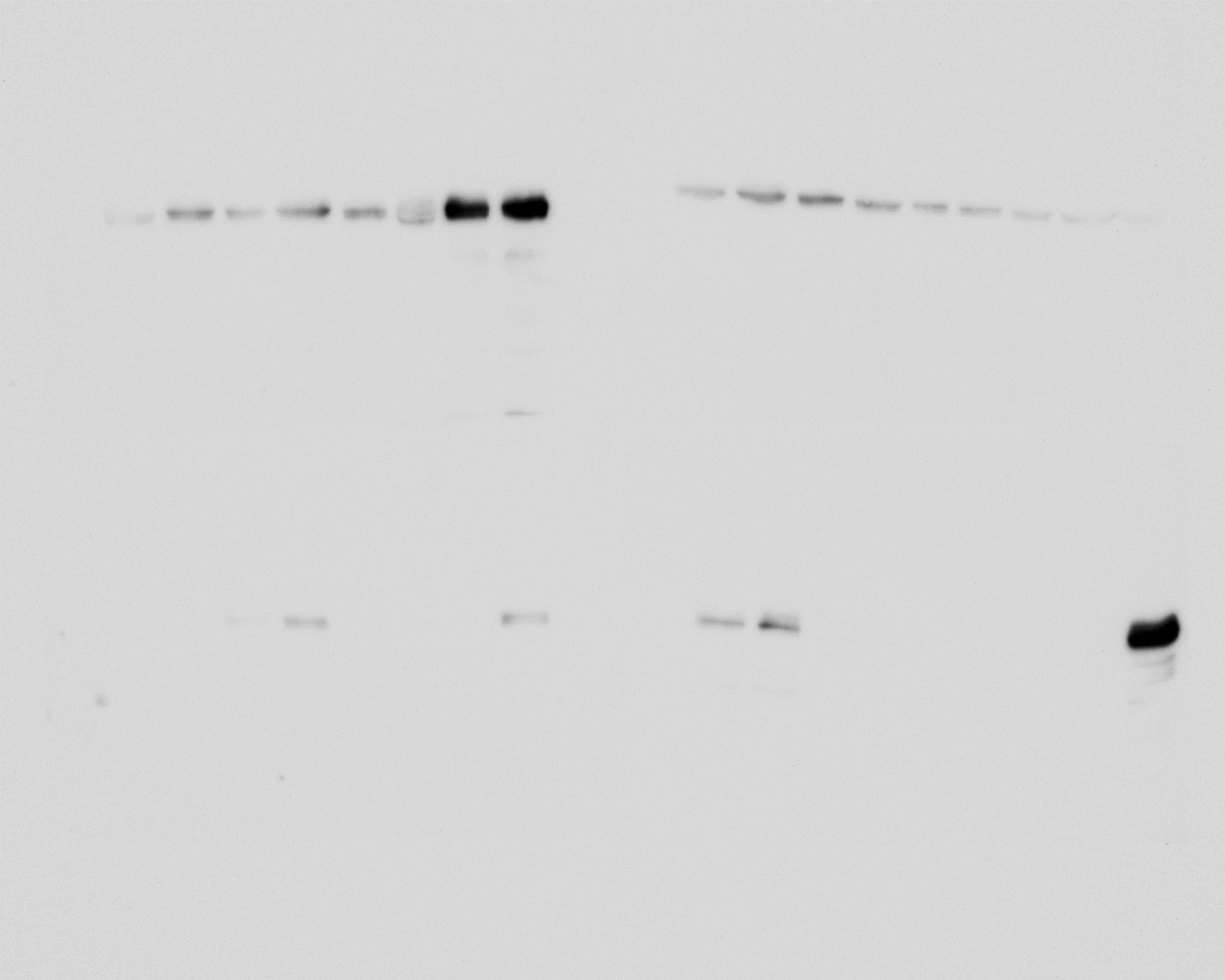

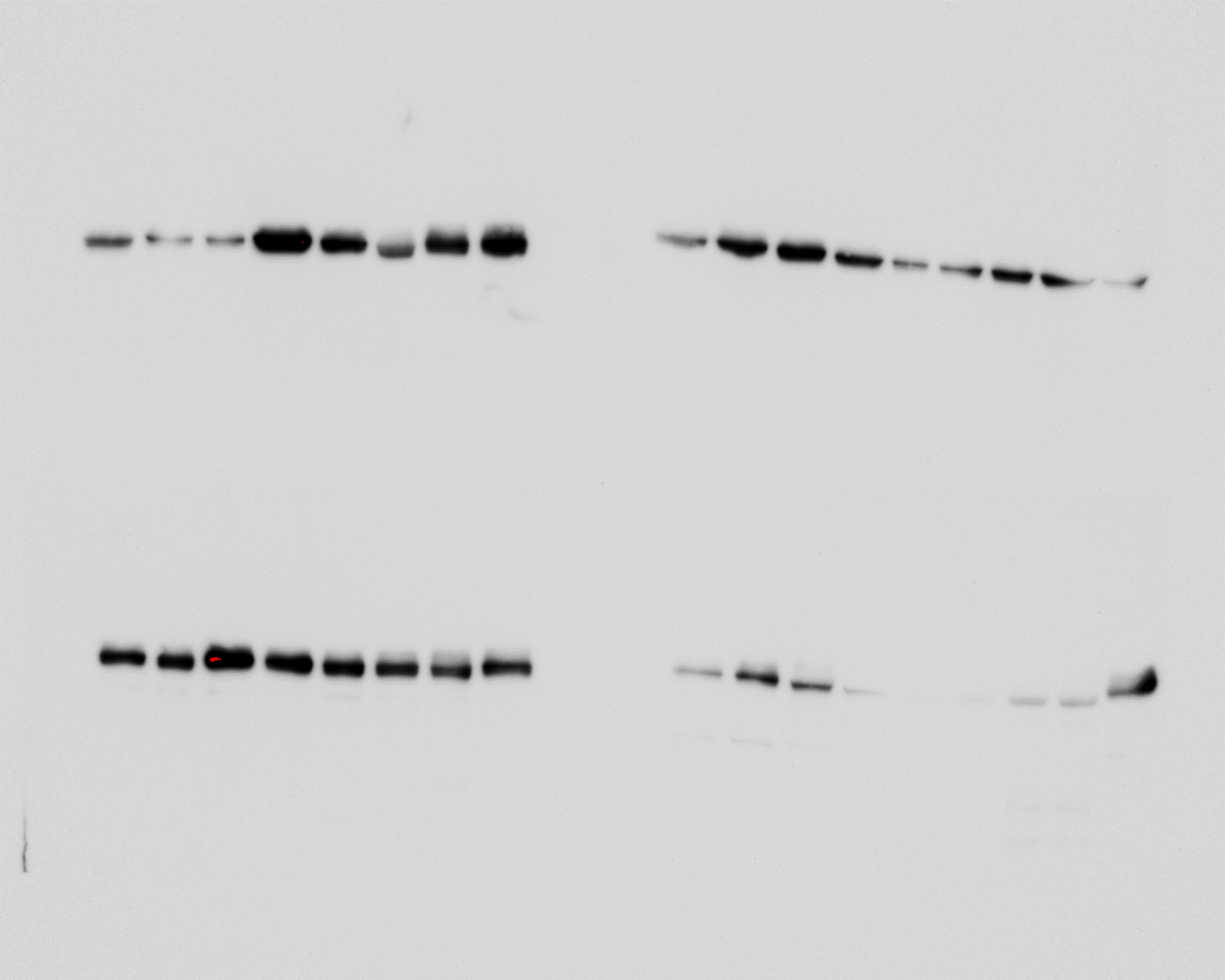


N1 LG NG

Actin -

AMPK-p -

AMPK -

3.1 16.9 16.0

5.8 12.7 11.5

**Supplementary Fig. 1 – Metabolic stress induces activation of AMPK in hOL.** hOL were treated for 2 days in optimal culture conditions (N1), low glucose (LG) and no glucose conditions (NG). Actin was used as loading control. Number correspond to the level of AMPK related to actin.


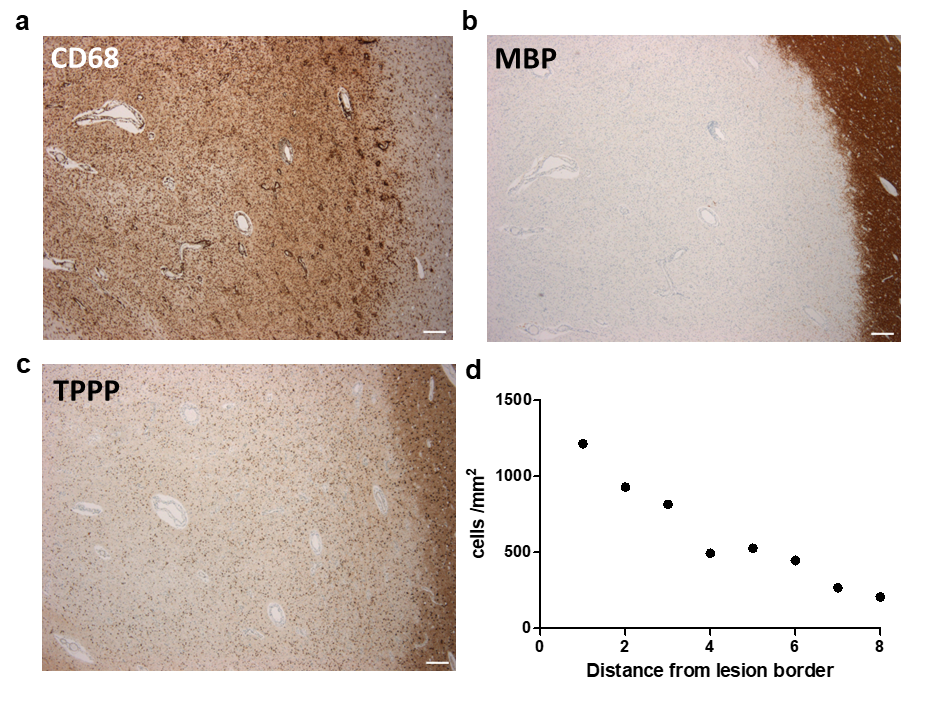


**Supplementary Fig. 2 Loss of hOL is intensified in the center of MS lesions. a-c** Sample images of an MS case showing adjacent normal appearing white matter (NAWM), lesion edge, and lesion center, immunolabeled with anti-CD68 (microglia/macrophages) (A), anti-MBP (myelin) (B), and anti TPP (OLs) antibodies (C). Scale bar correspond to 200 µm. **d** Number of hOLs from the border (0-2mm^2^) to the center (6-8mm^2^) of MS lesions.
